# Fibre Alignment and Void Assessment in Thermoplastic Carbon Fibre Reinforced Polymers Manufactured by Automated Tape Placement

**DOI:** 10.3390/polym13030473

**Published:** 2021-02-02

**Authors:** Tamer A. Sebaey, Mohamed Bouhrara, Noel O’Dowd

**Affiliations:** 1 Engineering Management Department, College of Engineering, Prince Sultan University, Riyadh 66833, Saudi Arabia; 2 Mechanical Design and Production Department, Faculty of Engineering, Zagazig University, Zagazig P.O. Box 44519, Sharkia, Egypt; 3 Saudi Aramco, Research & Development Center, Dhahran 31311, Saudi Arabia; MohamedmmBouhrara@aramco.com; 4 Irish Composites Centre (IComp), Bernal Institute, University of Limerick, V94 T9PX Limerick, Ireland; Noel.dowd@ul.ie

**Keywords:** thermoplastic composites, automated tape placement, fibre misalignment, voids, CT scan, circular distribution

## Abstract

Automated Tape Placement (ATP) technology is one of the processes that is used for the production of the thermoplastic composite materials. The ATP process is complex, requiring multiple melting/crystallization cycles. In the current paper, laser-assisted ATP was used to manufacture two thermoplastic composites (IM7/PEEK and AS4/PA12). Those specimens were compared to specimens that were made of thermoset polymeric composites (IM7/8552) manufactured while using a standard autoclave cycle. In order assess the quality, void content, fibre distribution, and fibre misalignment were measured. After manufacturing, specimens from the three materials were assessed using optical microscopy and computed tomography (CT) scans. The results showed that, as compared to the thermoset composites, thermoplastics that are manufactured by the ATP have a higher amount of voids. On the other hand, manufacturing using the ATP showed an improvement in both the fibre distribution inside the matrix and the fibre misalignment.

## 1. Introduction

Fibre-reinforced plastic (FRP) composites are extensively used in aerospace, submarine, automotive, and petrochemical industries for their high strength and stiffness to weight ratios, chemical stability, and ability to tailor strength and stiffness properties by tailoring the fibre orientation [1,2]. For more than half a century, most of the polymer composite parts were designed of thermoset matrix, due to their thermal stability and strength. During the last two decades, interest in thermoplastic composites has increased due to their superior properties, such as short processing times, long-term storage, ease of recycling, and impact properties [3,4,5]. The manufacturing of thermoplastic composite parts requires a heating process, either directly before the final moulding process, where a heat source plus a cool tool is used (non-isothermal processing), or in a hot mould (isothermal processing) [6]. Laser-assisted automated tape placement (ATP) is one of the non-isothermal processes that uses laser as a heat source to melt the thermoplastic matrix while tapes are laid-up on a tool, and then consolidated under a roller [7].

Many factors influence the final quality of the FRP, including resin viscosity, difference in thermal expansion coefficient, and control of processing parameters. Therefore, several types of defects can be found, such as porosity (void content), fibre waviness (misalignment), and poor distribution of fibres in the matrix, which results in resin-rich areas [8,9]. The effect of these defects on the mechanical properties of the composite parts is well documented in the literature [10,11,12]. During the manufacturing of FRP, if both pressure and application time are not optimized, voids could arise in the laminate [13]. The presence of voids reduces the interlaminar shear, longitudinal compressive, and transverse tensile strengths, in addition to inducing local stress concentrations, with consequent severe degradation of strength and stiffness in-service [12,14]. For aeronautical and other safety critical applications, the void content is limited to 1%, whereas, for other applications, 5% can be accepted, depending on the application [15].

The inhomogeneous distribution of fibres inside the polymer matrix results in areas with a lower volume of fibre. These areas are called resin-rich areas. The manufacturing technique [16,17] and the fibre architecture [18] are the main contributors to this lack of “perfect” uniform distribution. The resin rich areas are another source of stress concentration that need to be avoided for better quality of the manufactured composites [19]. In a numerical analysis, Ghayoor et al. [20] showed that, at the same fibre volume fraction, the failure initiation strain in the matrix is 20% lower for samples with resin rich areas when compared to samples without resin pockets.

Fibre misalignment is the third manufacturing defect that is taken into consideration in this paper. It results from the improper manufacturing parameters and thermal stress during the curing/crystallization [21]. The misalignment is considered as an initial fibre-kinking and, consequently, it directly affects the compression strength [22]. For a multidirectional laminate under general loading conditions, Fedulov et al. [23] numerically studied the effect of the ply misalignment in unidirectional (UD) composites and showed that it leads to a reduction of up to 49% in the laminate compressive strength. This effect was experimentally confirmed by Wilhelmsson et al. [24]. The fibre misalignment can be measured by many techniques, including ultrasonic array [25], mathematical representation of ellipse resulting from cutting the individual fibre [26], optical microscopy [27], and computed tomography (CT) scan [28].

The development of the manufacturing technology leads to improvements in the type and impact of the manufacturing defects. Most of the data that are reviewed are for thermoset-based composites and there are little data available on thermoplastic based composites [29]. In the current paper, the aim is to assess the quality of thermoplastic-based carbon fibre-reinforced composites manufactured using automatic controlled laser-assisted ATP. The parameters used in the assessments are the defects mentioned in the introduction; the measurement techniques are optical microscopy and CT scan.

## 2. Materials Manufacturing and Measurements

### 2.1. Materials and Manufacturing Techniques

Two thermoplastic-based composite systems are used in this study. The first one is IM7/PEEK, with poly(etheretherketone) as the polymeric matrix and IM7 carbon fibre. The second one is AS4/PA12, with polyamide 12 as the thermoplastic matrix and AS4 as the carbon fibre. Both of the material systems are in the form of tape (of the fibres embedded in the matrix) ready to be processed by the ATP. In addition, a thermoset-based composite is used as a reference, namely IM7/8552 in the form of prepreg (of the fibres embedded in the matrix), to be processed in an autoclave. The matrix and fibre properties can be found on the material data sheets [30,31,32]. Table 1 shows some of the characteristics of the three materials systems that were used in this study. Table 2 summarizes the properties of the three matrix systems, as per the materials data sheets.

The standard autoclave cycle was used to manufacture the IM7/8552 composites. Firstly, squares of 200 × 200 mm were cut from the prepreg and manually laid, with the corresponding stacking sequence. For this study, all of the specimens are designed to be ${\left[0_{2}/90_{4}\right]}_{S}$, with 12 layers in total. This stacking sequence was selected with a cluster of 8 layer of $90^{\circ }$ in the middle to be able to clearly see the fibre cross-section with the different scanning techniques. The idea behind the $0^{\circ }$ layers was to give strength to the samples while manufacturing and handling. The plies were laid and placed in an autoclave. The autoclave cycle consists of the following steps [32]: (1) apply full vacuum (1 bar), (2) apply 7 bar gauge autoclave pressure, (3) reduce the vacuum to a safety value of 0.2 bar when the autoclave pressure reaches approximately 1 bar gauge, (4) heat at 1–3 ${}^{\circ }$C/min to 110 ${}^{\circ }$C, (5) hold at 110 ${}^{\circ }$C for 60 min., (6) heat at 1–3 ${}^{\circ }$C/min to 180 ${}^{\circ }$C, (7) hold at 180 ${}^{\circ }$C for 120 min., (8) cool at 2–5 ${}^{\circ }$C/min, and (9) vent autoclave pressure when the component reaches 60 ${}^{\circ }$C. After curing, 30 mm were trimmed from all laminate edges using a numerically controlled diamond cutter with a hydraulic specimen holding mechanism.

The thermoplastic composites were manufactured by automated tape placement ATP. The machine uses a laser-assisted tape placement head (AFPT, GmbH) that was attached to a robot arm (Kuka, KR240 L210-2) and it is placed in a closed clean room. The manufacturing using the ATP starts by placing the tape in the right position through the robot arm. The laser beam melts, in situ, the laid tape and, consequently, a silicone consolidation roller applies pressure and cooling to finalise the process. The silicon roller applies pressure through a pneumatic cylinder. The roller is cooled externally using compressed air to avoid the tape to adhere the roller. Studies on the process parameters of the ATP can be found elsewhere [33,34]. Those processing parameters include: (a) the laser power, (b) the laser applied angle, (c) roller material and pressure, (d) tool temperature, (e) lay-down speed, and (f) roller temperature. For the current study, the processing parameters were selected based on the previous parametric study [35] and Table 3 shows the results.

**Figure 1:**
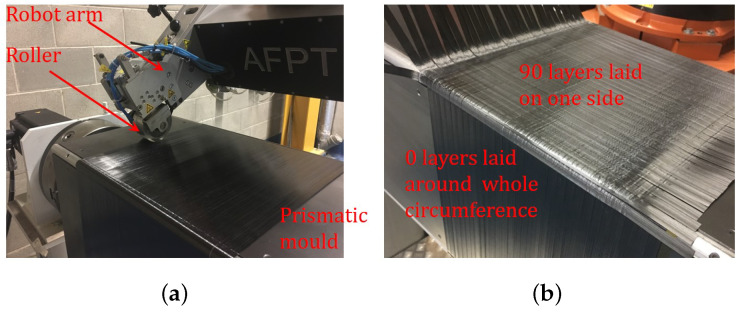
Using the prismatic mould with the robotic arm to manufacture the thermoplastic composite; (**a**) Laying the 0${}^{\circ }$. (**b**) Laying the 90${}^{\circ }$.

Securing the flatness of the plates manufactured while using this technology is a challenge, given that the plate is under in-situ thermal stresses through the whole manufacturing cycle. This thermal stress causes several deformations to the plate. A mechanical solution is proposed for solving this problem by manufacturing using a prismatic mould with square cross-section to lay the first two layers [0${}_{2}$] around the whole circumference. The next eight layers [90${}_{8}$] were laid on only one side of the square cross-section. Finally, the last two layers [0${}_{2}$] were applied to the whole circumference. Figure 1 shows the manufacturing details. The benefit of this procedure is to ensure a flat laminate surface that is maintained by the four [0${}_{2}$] layers surrounding the structure. After complete consolidation, the three non-effective sides of the mold were removed to leave a flat panel with the required stacking sequence.

Because of the multiple in situ melting/crystallization process during the manufacturing of thermoplastic composites using ATP, the resulting panel is not perfectly flat. In addition to panel flatness, the void content of ATP composite panels can be high [36]. To minimise void content and improve specimen flatness, two panels (one IM7/PEEK and one AS4/PA12) were subjected to autoclave cycles of 1 bar maximum pressure for three hours and maximum temperature of 400 ${}^{\circ }$C and 200 ${}^{\circ }$C, respectively. The cool down rate that was used for this process was 4 ${}^{\circ }$C/min This autoclave cycle melts the pre-manufactured (using ATP) panels under vacuum and pressure, and allows for it to solidify, which releases the thermal induced residual stresses and reduces the void volume. By adding these two panels, our test matrix was composed of a thermoset-based composite panel, two thermoplastic-based panels manufactured using ATP, and other two thermoplastic-based panels manufactured using ATP, and then treated using autoclave. In total, five conditions are tested.

In an earlier study, Rao et al. [37] studied the mechanical properties and damage mechanisms of the five conditions that were adopted in the current study through in-situ tensile loading. For the AS4/PA12 without autoclave treatment, the failure load and stress were 0.98 kN and 288 MPa, respectively. For the specimens with autoclave treatment, the failure load and stress were 1.48 kN and 363 MPa, respectively. This improvement in both the load carrying capacity and strength was governed by reducing the amount of voids and thickness reduction resulting from the compression in the autoclave cycle.

### 2.2. Measurements

Two non-destructive testing techniques were used to assess the quality of the composites. The first one was optical microscopy. Three specimens of 15 mm were cut from each panel and then mounted in silicon moulds of 50 mm diameter. The moulds were filled with epoxy resin (Transparent epoxy resin from Resineco) and left overnight for curing. After curing, the mounted specimens were grinded (with P600 for 5 min, P1200 for 10 min, and P2500 for 10 min) and then polished (with 0.3 microns polishing media for 30 min and 0.05 microns polishing media for 10 min) for the optical microscopy scans. The optical microscope (Axio imager MAT microscope from Zeiss) was used to monitor the specimens with different magnification factors (5×–50×). The images that were obtained from the optical microscope were processed using ImageJ software [38] to calculate the percentage of the area covered by the fibres and/or voids with respect to the total image area. The average of this value was taken as the fibre/void volume fraction. In addition, the tolerance in this measurement was used as a rubric to assess the fibre distribution i.e., the higher the tolerance means that the variation in the amount of fibre between the different images is high, which is an indication of the non-uniform distribution of the fibre inside the matrix.

The second tool used was the μCT scan, which was mainly used to assess the fibre misalignment. A total of nine scans were considered for each material. The values of the voltage and current of the X-ray were 80 kV and 87 μA, respectively. The images obtained were 924 × 924 pixels, with a pixel size of 0.36 μm. The result of this scan is a cylinder of 300 μm diameter and 300 μm length. Each scan resulted in 3201 projections (images), with an exposure time of 5 s, and specimen rotation of 360${}^{\circ }$. Each scan required approximately 270 min. to be completed. The images of the cross-section, taken during the previous step, were then reconstructed into a 3D shape using ImageJ software [39]. Slices, parallel to the fibre direction, were taken and the fibre pass was followed by segments of 30 μm each. The angles of these segments were measured, with reference to the principal direction, and they were collected for the same material and then plotted to show the distribution of the fibre misalignment. These data were fitted using the von Mises circular distribution as [40,41]:

(1) $$P\left(\alpha ,\mu ,\kappa \right)=\frac{1}{2\cdot \pi \cdot I_{0}\left(\kappa \right)}\cdot e^{\left(\kappa \cdot \cos\alpha -\mu \right)}$$

where α is the parameter being analysed, which is the misalignment in our study (in radians), μ represents the mean, at which the distribution is clustered around, and κ is the concentration parameter. $I_{0}$ is the modified Bessel function of order 0. For κ equals zero, the von Mises distribution is uniform. On the other hand, for small values of κ, the distribution is close to uniform. For a higher value of κ, the distribution becomes very concentrated regarding the angle μ. In our application, the higher the value of κ, the closer the fibres are to the zero angle, which represents an excellent alignment of the fibres to the desired angle.

## 3. Results and Discussion

### 3.1. Thermoset- vs. Thermoplastic-Based Composites

**Figure 2:**
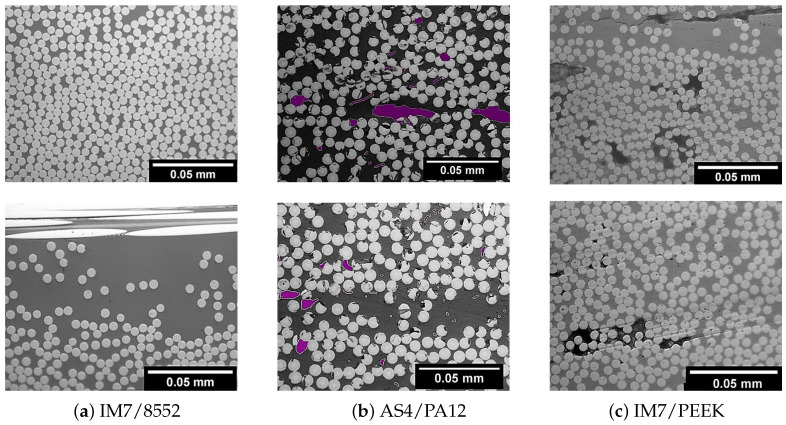
Sample of the optical microscope images that were obtained for the three material systems (Voids in AS4/PA12 are covered with purple for easy identification).

Figure 2 shows a sample of the images that were obtained from the optical microscopy scan for the thermoset- and the two thermoplastic-based composite panels. The first observation is the amount of voids. In the thermoset composites (Figure 2a), for more than 50 images, there are no visible voids, which reflects the well-established quality of the autoclave processing of the thermoset composites. This result can be justified by the vacuum and pressure applied to the specimen during the solidification process inside the autoclave. For the material that was manufactured with ATP (Figure 2b,c), the voids are obvious and appear to be a feature of thermoplastic composites manufactured by ATP. For the AS4/PA12, the voids are coloured with purple to enhance visibility. For the others, the voids are clear without changing the colour. These remarkable voids give evidence that the pressure that is applied by the roller is not enough and/or the flow of the matrix in the liquid state needs more control to fill in all of the areas inside the composites.

By phase separation on the ImageJ software, the amount of voids are estimated for both the IM7/PEEK and AS4/PA12 as 5.1${}^{\pm 0.6}$% and 5.1${}^{\pm 0.9}$%, respectively. With the same phase separation technique, the amount of fibres in each image is calculated and its area is divided by the image area in order to calculate the fibre volume fraction. The values of the fibre volume fraction measured for the three materials are $61^{\pm 16}$%, $55^{\pm 8}$%, and $53^{\pm 10}$% for the IM7/8552, AS4/PA12, and IM7/PEEK, respectively (Table 4). Those numbers are obtained from at least 50 images for each material. These numbers give an indication on the amount of fibre in the matrix, although, due to the high tolerance in the measurements, other techniques [42,43] give higher precision. The numbers showed that the specimens manufactured by the autoclave (the thermosets) contained the same fibre volumes fraction, as delivered by the prepreg manufacturer (see Table 1), which reflects the quality of the autoclave process and minimum void contents. On the other hand, the results for the volume fraction of the thermoplastic material is about 10% lower than the desired values, as per the material data sheets, as in Table 1. This is highly affected by the void content, as it increases the total plate volume.

The tolerance (scatter) in the measurement of the fibre volume fraction is used as an indication of the distribution of the fibres in the matrix. A higher variation corresponds to higher deviation of the volume % in between the images taken for the same materials. The values of the tolerance are 16%, 8%, and 10 % for the IM7/8552, AS4/PA12, and IM7/PEEK, respectively. This result indicates that the distribution of the fibres in specimens manufactured by ATP is better than that of the autoclave, which results in more resin rich areas in the thermoset composites.

**Figure 3:**
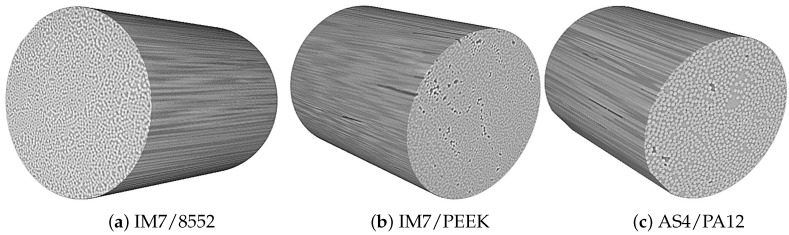
Samples of the constructed computed tomography (CT) scans for the entire test campaign.

Figure 3 shows samples of the CT scans that were obtained for each material. The first observation is the amount of voids that appears in IM7/PEEK and AS4/PA12. This method could also be used for the void content measurements [44], but, due to the high cost and limited sample size, the authors decided to proceed with the data from the optical microscopy. This observation is correlated to the lower volume fraction obtained for both of the thermoset-based composites.

**Figure 4:**
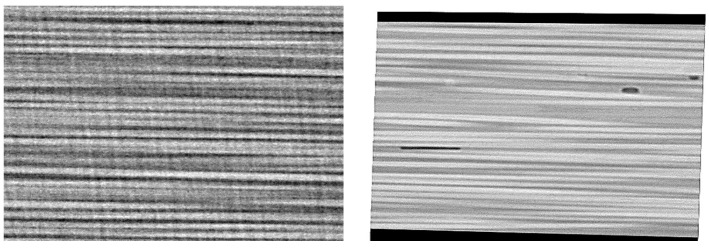
Samples of the two-dimensional (2D) images obtained through the fibre direction from the three-dimensional (3D) images resulting from the CT scan.

**Figure 5:**
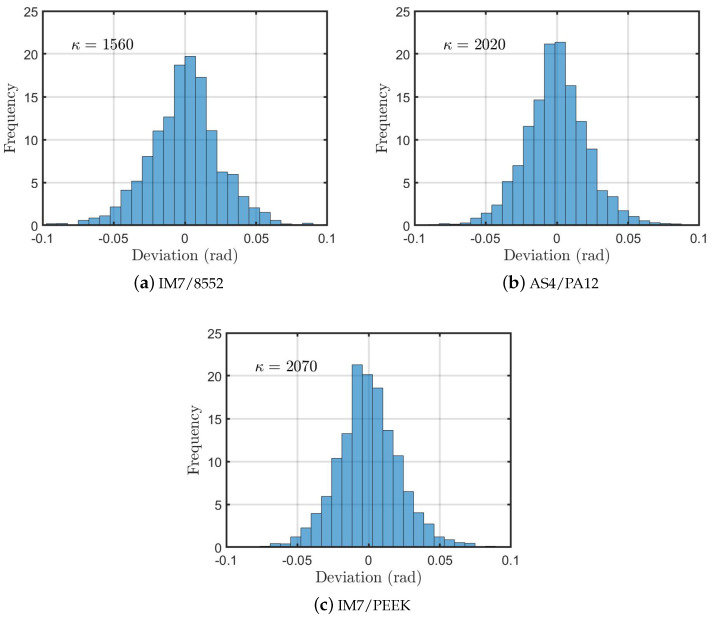
Fibre misalignment distribution for the IM7/8552, AS4/PA12, and IM7/PEEK, and the corresponding concentration parameter κ.

The main result of the CT scans is the fibre misalignment measurement. From the 3D images, sections at different locations that are parallel to the fibre direction were analysed in order to determine the fibre waviness/deviation from zero. Samples of the 2D sections are shown in Figure 4. Using Image-J, the footprint of each individual fibre was followed to measure the angle of the single fibre at an average of 8–10 different locations. The result of this process was a set of angles of almost 200 data points for each 2D image. Of the three 3D images presented in Figure 3, at least 4000 data points were considered to plot the distribution of the fibre waviness for each material. Figure 5 shows the distributions for the materials under consideration.

The results for the different material systems are analysed while using the circular toolbox [41] to find the von Mises concentration parameter (κ). The value of κ is listed on each distribution, Figure 5. Again, the statistical data that were obtained from fitting the measured misalignment angles with the von Mises distribution confirm that the samples with the highest fibre alignment are those of the IM7/PEEK (thermoplastic matrix) with a concentration parameter κ = 2070. This value is close to the one that was obtained for the AS4/PA12 (κ = 2020), which suggests that this improved alignment is related to the manufacturing process, rather than being a material-related issue. When compared to the thermoplastic-based composites, the thermoset composite IM7/8552 shows a lower value of the concentration parameter (κ = 1560). In summary, the standard autoclave cycle that is used in the processing of thermosets seems to lead to some local waviness/misalignment of individual fibres due to the applied pressure. On the other hand, in the ATP process, the composite is being solidified, while the individual fibres are in tension. This provides a constraint on the fibres, which reduces the misalignment.

### 3.2. Thermoplastics Treated by Autoclave after ATP Manufacturing

**Figure 6:**
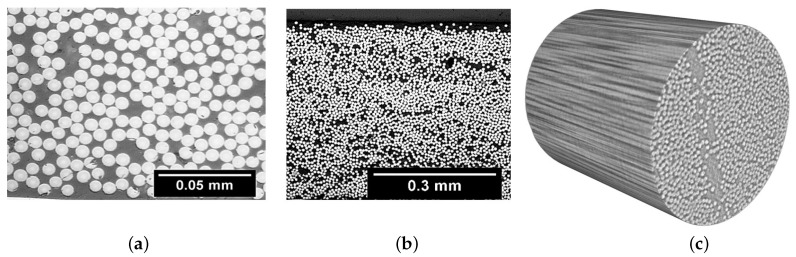
Inspection of AS4/PA12; (**a**) A high magnification. (**b**) A low magnification. (**c**) 3D CT image.

**Figure 7:**
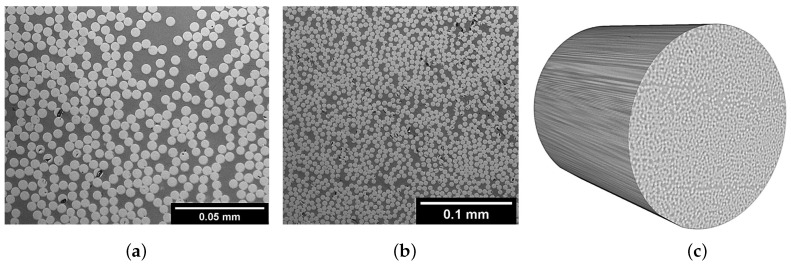
Inspection of IM7/PEEK; (**a**) A high magnification. (**b**) A low magnification. (**c**) 3D CT image.

The samples of the images obtained from the specimens treated by the autoclave, after being manufactured by ATP, are shown in Figure 6 and Figure 7 for the AS4/PA12 and IM7/PEEK, respectively. Autoclave post processing of the AS4/PA12 (Figure 6) shows a significant impact on the void content (both size and number). No large voids appear, as compared to the ones that are shown in Figure 2. Other feature is the uniform fibre distribution. The observation is the same for the images in Figure 7 when compared to the images without treatment in Figure 2 for the IM7/PEEK composites. This finding of the lack of voids is also confirmed from the 3D CT scans for both materials.

The measured fibre volume fraction is 59${}^{\pm 4}$% and 60${}^{\pm 7}$% for the AS4/PA12 and IM7/PEEK, respectively. The values of the fibre volume fraction are closer to the ones that were supplied by the supplier (Table 1), as compared to the values that were obtained without autoclave treatment. In addition, deviations are less for those specimens treated by autoclave. This result implies that the fibre distribution is better in the treated specimens. This improvement can be justified by the pressure that is applied to the thermoplastic specimens in the autoclave that squeezes the fibres in the liquid matrix.

**Figure 8:**
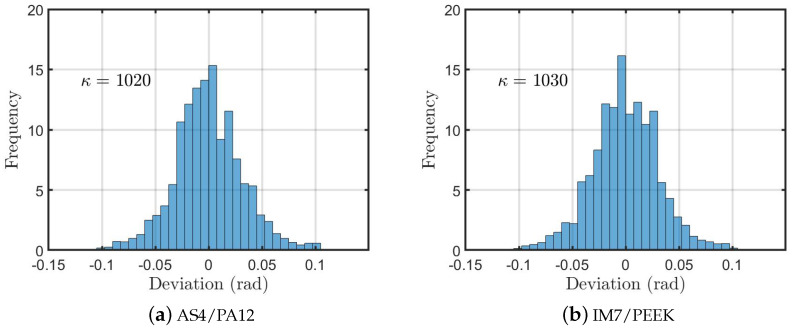
Fibre misalignment distribution for the samples treated by autoclave after being manufactured by ATP and the corresponding concentration parameter κ.

Figure 8 represents the misalignment distribution obtained from the specimens post processed by autoclave after ATP manufacturing. The readings of the current samples show higher values of misalignment angle with less concentration around the zero degree (the ideal UD lamina) as compared to the samples without post processing (Figure 5). As a result, the corresponding values of the concentration parameters κ are smaller than that of the specimens without treatment, which implies more fibre misalignment/waviness.

In the autoclave post-processing, the thermoplastic matrix is fully melted and then pressed in order to minimize the voids. As a side effect ot this process, the fibres experience micro and sometimes macro unconstrained movement. This justifies the smaller values of the concentration parameter, which is a result of the poor alignment of the fibres. This result shows that, although it reduces the void content, the autoclave cycle has a negative effect on the fibre alignment.

## 4. Conclusions

This paper examines the quality of composite parts that are manufactured by the automated tape placement (ATP) technology. Two thermoplastic composites manufactured by ATP were used herein, namely IM7/PEEK and AS4/PA12. For reference, the authors used the well-known thermoset aeronautical composites, IM7/8552, manufactured by standard autoclave cycle. The parameters that were used in the assessment were the distribution of the fibre inside the matrix, the void contents, and the misalignment of the fibres. The methodology that was used for the assessment consists of optical microscopy scans and CT scans followed by statistical analysis.

The results showed that using the ATP with the prescribed manufacturing parameters results in a higher volume fraction of voids, when compared to the autoclave cycle used in the manufacturing of the thermoset-based composites. This is a challenge to be addressed in order to fully benefit from the huge capability of this automated manufacturing technique.

On the other hand, as compared to the epoxy-based composite (IM7/8552), the thermoplastic composites (IM7/PEEK and AS4/PA12) showed improved quality in terms of the distribution of the fibres inside the matrix as well as the fibre misalignment. The crystallization in ATP occurs while the fibres are under tension, which justifies this improved fibre misalignment in the automated tape placement of thermoplastic composites.

The specimens that were treated by autoclave cycle with 1 bar pressure and a temperature higher than the melting temperature showed improvements in the void contents and the fibre distribution in the matrix. However, the autoclave treatment also results in a degradation in the fibre misalignment, as the process melts the polymeric matrix and allow under pressure solidification while the individual fibres are unconstrained. 

## Figures and Tables

**Table 1:** Characteristics of the IM7/PEEK, AS4/PA12, and IM7/8552 CFRP composites.

Material	Fibre Volume Fraction	Ply Thickness	Density	Tape/Prepreg Width
	%	mm	g/cm${}^{3}$	mm
IM7/PEEK	60.0	0.14	1.588	12
AS4/PA12	60.0	0.133	1.48	12
IM7/8552	60.0	0.131	1.57	400

**Table 2:** Characteristics of the three matrix materials, as per the material data sheets [30,31,32].

Property	PEEK	PA12	8552 Epoxy Resin
Trade name	VICTREX PEEK 150UF10	VESTOSINT 2159	HexPly 8552
Supplier	Suprem	Suprem	Hexcel
Melting point (${}^{\circ }$C)	343	184	—
Glass transition (${}^{\circ }$C)	143	49	200
Density (g/cm${}^{3}$)	1.30	1.02	1.30
Tensile strength (MPa)	100	73	121
Tensile Modulus (GPa)	4.00	1.96	4.67

**Table 3:** Manufacturing parameters that are used by the ATP to manufacture IM7/PEEK and AS4/PA12 CFRP composites.

Lay-Down Speed	Target Temperature	Roller Material	Pressure
12 m/min	420 ${}^{\circ }$C for IM7/PEEK	Silicone	1.2 Bar
	210 ${}^{\circ }$C for AS4/PA12		

**Table 4:** Measured void contents and volume fraction of the three composite materials.

Property	IM7/PEEK	AS4/PA12	IM7/8552
Void contents (%)	5.1${}^{\pm 0.6}$	5.1${}^{\pm 0.9}$	0.0
Fibre volume fraction (%)	$53^{\pm 10}$	$55^{\pm 8}$	$61^{\pm 16}$

## Data Availability

The data presented in this study are available on request from the corresponding author.
